# Respiratory viral infections detected by multiplex PCR among pediatric patients with lower respiratory tract infections seen at an urban hospital in Delhi from 2005 to 2007

**DOI:** 10.1186/1743-422X-6-89

**Published:** 2009-06-26

**Authors:** Preeti Bharaj, Wayne M Sullender, Sushil K Kabra, Kalaivani Mani, John Cherian, Vikas Tyagi, Harendra S Chahar, Samander Kaushik, Lalit Dar, Shobha Broor

**Affiliations:** 1 Department of Microbiology, All India Institute of Medical Sciences, Ansari Nagar, New Delhi, 110029, India; 2 Department of Pediatrics, University of Alabama at Birmingham, Alabama, 35294-0011 USA; 3 Department of Pediatrics, All India Institute of Medical Sciences, Ansari Nagar, New Delhi, 110029, India; 4 Department of Biostatistics, All India Institute of Medical Sciences, Ansari Nagar, New Delhi, 110029, India

## Abstract

**Background:**

Acute lower respiratory tract infections (ALRI) are the major cause of morbidity and mortality in young children worldwide. Information on viral etiology in ALRI from India is limited. The aim of the present study was to develop a simple, sensitive, specific and cost effective multiplex PCR (mPCR) assay without post PCR hybridization or nested PCR steps for the detection of respiratory syncytial virus (RSV), influenza viruses, parainfluenza viruses (PIV1–3) and human metapneumovirus (hMPV). Nasopharyngeal aspirates (NPAs) were collected from children with ALRI ≤ 5 years of age. The sensitivity and specificity of mPCR was compared to virus isolation by centrifugation enhanced culture (CEC) followed by indirect immunofluorescence (IIF).

**Results:**

From April 2005–March 2007, 301 NPAs were collected from children attending the outpatient department or admitted to the ward of All India Institute of Medical Sciences hospital at New Delhi, India. Multiplex PCR detected respiratory viruses in 106 (35.2%) of 301 samples with 130 viruses of which RSV was detected in 61, PIV3 in 22, PIV2 in 17, hMPV in 11, PIV1 in 10 and influenza A in 9 children. CEC-IIF detected 79 viruses only. The sensitivity of mPCR was 0.1TCID_50 _for RSV and influenza A and 1TCID_50 _for hMPV, PIV1, PIV2, PIV3 and Influenza B. Mixed infections were detected in 18.8% of the children with viral infections, none detected by CEC-IIF. Bronchiolitis was significantly associated with both total viral infections and RSV infection (p < 0.05). History of ARI in family predisposed children to acquire viral infection (p > 0.05).

**Conclusion:**

Multiplex PCR offers a rapid, sensitive and reasonably priced diagnostic method for common respiratory viruses.

## Background

Acute respiratory tract infections (ARI) are a leading cause of morbidity and mortality in children worldwide [1] accounting for about 30% of all childhood deaths in developing world [2]. Viruses account for 50–90% of acute lower respiratory tract infections (ALRI) in young children [3] with respiratory syncytial virus (RSV), parainfluenza viruses (PIV), influenza viruses A and B and human metapneumoviruses (hMPV) being most commonly identified [4-6].

Respiratory infections caused by above said viruses usually present with clinical features that are nearly indistinguishable [7]. The increased sensitivity of polymerase chain reaction (PCR) over conventional methods for the diagnosis of respiratory viral infections has been established previously [8]. However, organism-specific RT-PCR assays which require separate amplification of each virus under investigation are resource intensive, time consuming and labor intensive [9].

Multiplex PCRs (mPCR) detect multiple organisms in a single assay and are available either as commercial assays [9-12] or in-house assays [4,5,13-17]. Majority of the in-house mPCR assays have not included recently discovered respiratory pathogens and require validation of results by post PCR hybridization or semi/nested PCR which make the assay cost ineffective and increases chances of cross contamination. Commercially available mPCR assays are expensive and require dedicated instrumentation [18].

We developed a simplified and economical multiplex PCR assay without any post PCR hybridization/nested PCR steps for the detection of seven major respiratory viruses.

(This material was presented in part at the 7^th ^Asia Pacific Congress of Medical Virology held at New Delhi, India in November 2006 and Options for the Control of Influenza VI held at Toronto, Canada in June 2007.)

## Results

The primer set designed for PIV1 failed to amplify PIV1 RNA after repeated attempts. The primer set published by Osiowy in 1998 [17] was found to amplify PIV1 N gene successfully.

### Standardization of cDNA synthesis

Ten Units of the AMV-RT enzyme, 500 ng of random hexamer primer (PdN_6_), 500 μM dNTP concentration and 8 U of RNAsin were found to be optimal for 25 μl cDNA synthesis.

### Standardization of multiplex PCR

**Figure 1:**
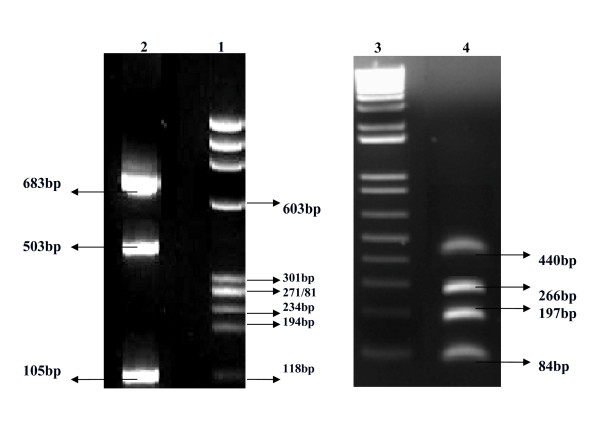
**Standardization of two tube multiplex PCR for RSV, Influenza A&B viruses in first tube and PIV1–3 and hMPV in the second tube**. Lane 1: Marker Ø X174 (*Hae III *digested). Lane 2: Amplicon forRSV showing band of 683 bp, Influenza A of 105 bp, Influenza B of 503 bp. Lane 3: Marker 100 bp ladder. Lane 4: Amplicon for PIV1 showing band of 84 bp, PIV2 of 197 bp, PIV3 of 266 bp, and hMPV of 440 bp.

All seven sets of primers when combined led to mispairing and nonspecific amplification. After trying different combinations, it was observed that RSV, Influenza A and B viruses in one set and PIV1–3 and hMPV in another set gave specific amplification for each virus (Figure 1).

### Optimized reagent and PCR cycling conditions for first and second tube multiplex PCR

The optimized reagent concentrations for each tube were 25 pM of each primer, 400 μM of dNTPs, 2 mM MgCl_2 _and 6 U of Taq polymerase enzyme. The optimized cycling conditions for both tubes were: 94°C for 3 min followed by 35 cycles of 94°C for 1 min, 55°C for 1 min (53°C for 1 min for second tube) and 72°C for 1 min. Final extension was done at 72°C for 10 min for first tube and 7 min for second set.

### Sensitivity and specificity of multiplex PCR

The sensitivity of detection by two tube multiplex PCR was 0.1TCID_50 _for RSV, Influenza A and 1TCID_50 _for hMPV, PIV1, PIV2, PIV3 and Influenza B. There were no non-specific amplification products against RNA from heterologous sources.

### Detection of seven respiratory viruses in clinical samples

#### Study group

Three hundred and one children from OPD and ward with ALRI were enrolled in the study. Of the 166 children seen in the outpatient department, 137 (82.5%) had ALRI, 29 (17.5%) had severe ALRI and none had very severe ALRI (as per WHO classification). Of the 135 children admitted to pediatric ward, 35 (26%) had ALRI, 92 (68%) had severe ALRI and 8 (6%) had very severe ALRI (Table 1). More number of children with ALRI were seen in the OPD as compared to pediatric ward (p < 0.05, Pearson Chi square test). However, for severe ALRI, more children were admitted than seen in OPD (p < 0.05, Pearson Chi square test).

**Table 1:** Distribution of children with ALRI/Severe ALRI/very severe ALRI from OPD or pediatric ward

Site	Clinical presentation			Total
		
	ALRI/172 (%)	Severe ALRI/121 (%)	Very Severe ALRI/8 (%)	
OPD	137 (79.6)^a^	29 (24)^b^	0	166
Pediatric Ward	35 (20.4)^a^	92 (76)^b^	8 (100)	135
Total	172	121	8	301

^a, b ^p value ≤ 0.05, Pearson chi square test

All the 301 children enrolled in the study were in the age range of 1–72 months with the median age of 11 months. The mean of their age was 15.6 ± 14 months. Among them 217 were males and 84 were females (Male: Female ratio = 2.6:1). It was observed that there was no significant difference between the age range of children with ALRI or severe ALRI from OPD or Ward (p > 0.05).

### Detection of seven respiratory viruses in children with ALRI

Of the 301, 106 children (35.2%) had viral infections and were positive for 130 respiratory viruses. Of these 106 children with ALRI, 64 presented to the OPD and 42 were admitted to the ward. Of the 64 children who presented to OPD, 52 had ALRI and 12 had severe ALRI. Of the 42 children who were admitted, 8 had ALRI, 31 had severe ALRI and 3 had very severe ALRI (Table 2).

**Table 2:** Distribution of children with ALRI/Severe ALRI/very severe ALRI from OPD or pediatric ward positive for different respiratory viruses

Site	Clinical presentation			Total
		
	ALRI/total ALRI (%)	Severe ALRI/total severe ALRI (%)	Very Severe ALRI/number (%)	
OPD	52/60 (81.3)	12/43 (27.9)	0	64
Pediatric Ward	8/60 (18.7)	31/43 (70.1)	3/3 (100)	42
Total virus positive	60	43	3	106

In 106 children in whom respiratory viruses were detected, the age range was 1–72 months with a median of 12 months and the mean age was 15.8 ± 13.8 months. In the PCR negative group, the age range was 1–61 months with a median of 10 months and the mean age was 15.5 ± 14.1 months. This difference was not statistically significant between the two groups. There was no significant difference in the male female ratio between the two groups.

**Figure 2:**
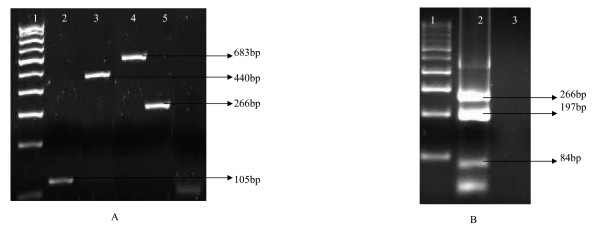
**Application of two tube multiplex PCR on clinical samples**. **Panel A**. Lane 1: 100 bp DNA ladder. Lane 2: Clinical sample showing amplicon of 105 bp for FLU A. Lane 3: Clinical sample showing amplicon of 683 bp for RSV. Lane 4: Clinical sample showing amplicon of 440 bp for hMPV. Lane 5: Clinical sample showing amplicon of 266 bp for PIV3. Lane 3: Negative clinical sample. **Panel B**. Lane 1: 100 bp DNA adder. Lane 2: Clinical sample showing mixed infection of PIV1 (84 bp), PIV2 (197 bp) and PIV3 (266 bp).

RSV was detected in 61, PIV3 in 22, PIV2 in 17, hMPV in 11, PIV1 in 10 and Influenza A in 9 children respectively (Table 3). Figure 2 shows detection of single and mixed infections in some samples on which two tube multiplex PCR was applied. Of these, 86 were single virus infections and mixed infections were seen in 20 children (18.8% of the 106 children). Nested PCR for RSV identified the presence of RSV B in all 61 samples. Of the single infections, RSV comprised 50, hMPV 9, Influenza A and PIV3 8 each, PIV2 6 and PIV1 5.

**Table 3:** Virus identifications in children with ALRI detected positive for viral infections by multiplex PCR

**Viruses detected by mPCR**	**Number of specimens**
RSV	50
Influenza A	8
PIV1	5
PIV2	6
PIV3	8
hMPV	9
PIV2+PIV3	6
RSV+PIV2+PIV3	3
RSV+PIV3	3
RSV+PIV1	3
RSV+PIV2	1
PIV3+INFA	1
PIV1+PIV2+PIV3	1
RSV+hMPV	1
hMPV+PIV1	1

TOTAL	106

It was seen that the percentage of virus detections by multiplex PCR were higher in the children with ALRI seen as outpatients (37.2%) as compared to those admitted to the ward (22.8%). Similarly, in the children with severe ALRI, seen as outpatients were higher percentage was positive for viruses (45%) as compared to those admitted to the ward (33.7%). It was observed that of all the mixed infections, 5.8% of them had ALRI whereas 7.8% of them had severe and very severe ALRI. Although severe ALRI was seen in higher number of children with mixed infections as compared to those with ALRI with mixed infections, the difference between the groups was not statistically significant.

### Co-relation between multiplex PCR and tissue culture

The "gold standard" isolation in tissue culture by CEC-IIF detected 79 (61%) viruses as compared to 130 by multiplex PCR. CEC-IIF could not detect the presence of viruses in samples with mixed infections (data not shown). The sensitivity, specificity and likelihood ratio between the two assays is shown in Table 4.

**Table 4:** Validity of multiplex PCR in comparison to CEC-IIF for the detection of respiratory viruses in children with ALRI

**Results (RT-PCR/CEC-IIA)**	RSV	INF A	PIV1	PIV2	PIV3	hMPV
**+/+**	27	8	5	10	18	11
**+/-**	34	1	5	7	4	0
**-/-**	274	293	296	291	283	290
**-/+**	0	0	0	0	0	0
**Total samples**	301	301	301	301	301	301
**Sensitivity (%)**	100	100	100	100	100	100
**Specificity (%)**	88.9	99.6	98.3	97.6	98.6	100
**Likelihood ratio (positive)**	9	250	59	42	71	-

### Temporal distribution of respiratory viruses

**Figure 3:**
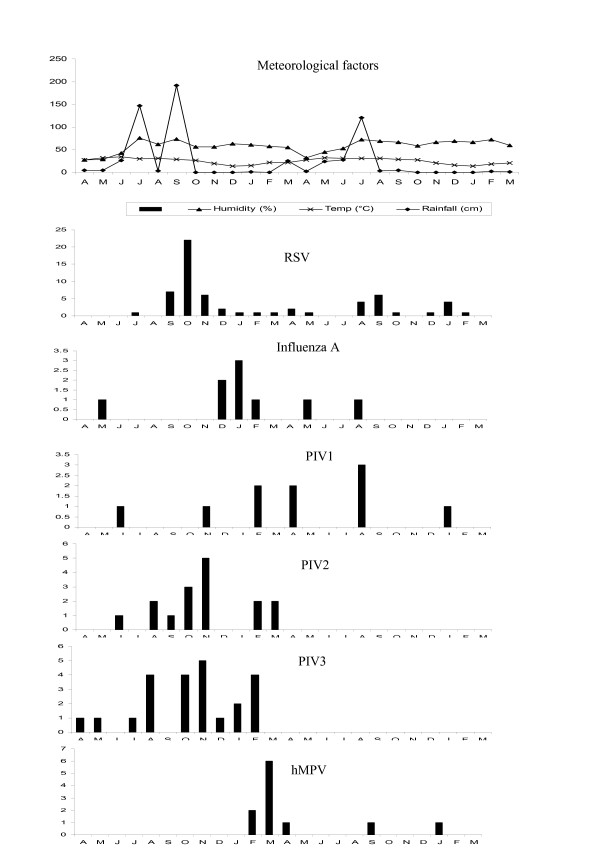
**Monthly distribution of ALRI causing viruses detected during the study**.

The number of RSV infections increased during late fall and peaked between October and January during the first year of the study. During the next year of the study, the distribution of RSV was scattered. PIVs were detected during the first year of the study, influenza A in winter months and hMPV in spring season (Figure 3).

### Cost effectiveness of the multiplex PCR assay

The cost per sample detected by two tube multiplex PCR assay was USD16 (RNA extraction USD6, cDNA synthesis USD2.5 and two tube multiplex PCR USD4.5, equipment and personnel cost USD3) as compared to the cost per sample by culture being USD24 (Sample collection USD2, sample processing USD2, inoculation of sample on to 3 different cell lines USD8, indirect immunofluorescence USD3, visualization under fluorescent microscope USD3, equipment and personnel cost USD6).

### Clinical symptoms

The clinical features, demographics and risk factors of children with viral infections and RSV alone were compared with the virus negative group (Table 5, 6). It was observed that significantly higher number of children below 12 months of age had RSV infection. Children presenting with preceding bronchiolitis were significantly associated with total viral infections and RSV infection (p < 0.05). Runny nose was significantly present in children with RSV infection (p < 0.05). Among risk factors, ARI in family was found to be associated with virus positive children (p < 0.005).

**Table 5:** Children with ALRI positive and negative for respiratory viruses by multiplex PCR

**Variables**	**Virus positive (n = 106)**	**Virus negative (n = 195)**	**p value**	**OR (95% CI)**
Median age (Mo)	12 (1–72)	10 (1–61)	0.36	-
Sex M:F	74:32 (2.3:1)	143:52 (2.8:1)	0.51	1.2 (0.68, 2.1)
**Clinical symptoms**				
Cough	102	183	0.43	3.1 (0.99, 1.25)
Difficulty in breathing	70	113	0.17	1.4 (0.84, 2.4)
Runny nose	50	70	0.05	1.6 (0.96, 2.6)
Sore throat	5	2	0.10	4.8 (0.76, 51.2)
Fever	88	159	0.74	1.1 (0.57, 2.2)
Hoarseness	6	6	0.27	1.9 (0.49, 7.3)
Asthma	7	7	0.23	1.9 (0.55, 6.5)
Grunting	3	5	0.99	1.1 (0.17–5.8)
Nasal flaring	13	18	0.40	1.4 (0.59–3.1)
Stridor	3	5	0.99	1.1 (0.17–5.8)
Chest indrawing	40	80	0.57	0.87 (0.52–1.5)
Cyanosis	5	7	0.63	1.3 (0.32, 5.0)
Recurrent pneumonia	7	15	0.03	4.5 (1.1, 27.6)
Pneumonia	84	151	0.71	1.1 (0.60, 2.1)
**Bronchiolitis**	**52**	**42**	**0.001**	**3.5 (2.0, 6.0)**
**Risk factors**				
**ARI in family**	**51**	**62**	**0.005**	**1.9 (1.2, 3.3)**
Prematurity	14	13	0.05	0.87 (0.41, 1.8)
Smokers in family	36	67	0.94	0.98 (0.58, 1.7)
Co-morbidity	19	66	0.003	0.43 (0.23, 0.78)

**Table 6:** Children with ALRI positive and negative for RSV by multiplex PCR

**Variables**	RSV positive (n = 50)*	**Virus negative (n = 195)**	**p value**	**OR (95% CI)**
Median age (Mo)	10.5 (2–48)	10 (1–61)	0.60	-
**Less than 12 months**	**38**	**115**	**0.027**	**2.2 (1.1, 4.5)**
Sex M:F	35:15 (2.3:1)	143:52 (2.8:1)	0.63	1.2 (0.55, 2.4)
**Symptoms**				
Cough	49	183	0.47	3.2 (.45, 140.1)
Difficulty in breathing	30	113	0.79	1.1 (0.55, 2.2)
**Runny nose**	**32**	**70**	**0.001**	**3.2 (1.6, 6.4)**
Sore throat	0	2	0.99	-
Fever	44	159	0.27	1.7 (0.64, 5.1)
Hoarseness	4	6	0.12	2.7 (0.54, 12.0)
Asthma	4	7	0.24	2.3 (0.49, 9.6)
Grunting	2	5	0.63	1.6 (0.15, 10.0)
Nasal flaring	5	18	0.86	1.1 (0.30, 3.3)
Stridor	2	5	0.63	1.6 (0.15, 10.0)
Chest indrawing	19	80	0.69	0.88 (0.44, 1.7)
Cyanosis	0	7	0.35	-
Recurrent pneumonia	3	15	0.99	4.1 (0.53, 31.2)
Pneumonia	38	151	0.82	0.92 (0.43, 2.1)
**Bronchiolitis**	**41**	**42**	**0.001**	**16.6 (7.1, 41.4)**
**Risk factors**				
ARI in family	23	62	0.06	1.8 (0.92, 3.6)
Prematurity	6	13	0.20	1.9 (0.56, 5.7)
Smokers in family	20	67	0.45	1.3 (0.63, 2.5)
Co-morbidity	5	66	0.001	0.22 (0.06, 0.59)

* episodes of co-infection with other viruses were excluded

## Discussion

The development of multiplex PCR for the detection of respiratory viruses as a rapid, sensitive and time saving technique has not gained priority in India even though ~0.5 million children die each year in this country due to ALRI each year, accounting for one fourth of the 1.9 million global ALRI deaths [19-21]. Among all the major ALRI causing viruses namely RSV, PIVs and influenza viruses A and B, the presence of RSV has been documented to be the most commonly identified pathogen followed by PIV3 [22]. In the present study, we standardized and evaluated an economical two-tube multiplex PCR assay devoid of any further confirmatory steps. The present assay reagents costs were mere USD16/reaction in contrast to USD90/reaction [18] reported for a commercial assay. The sensitivity of our multiplex PCR assay was similar or better than previously described mPCR assays for these viruses [5,16,17,23,24]. We did not make direct comparisons of the performance of the different assays in our laboratory.

In the present study we could culture majority of the viruses detected by mPCR with the exception of RSV which is known to be highly thermolabile [25]. The detection rate of viruses was similar to detection rate reported earlier [16,17,24,26]. It was observed that a higher proportion of virus positive children presented to the OPD than the ward, similar to a study from Taiwan [26] and could be due to the fact that the patients present earlier after onset of symptoms to the OPD as compared to getting admitted to the Ward. However, this could also be because severe disease is more likely to be admitted to the hospital and caused by bacteria than virus [27]. A higher proportion of males were found to have infection with respiratory viruses as compared to females as reported earlier [28,29].

RSV was most commonly identified viral pathogen similar to previously described viral identifications by mPCR [16,17,28,29]. PIVs were the second most frequently identified pathogens [29] followed by hMPV [22,28-30] and Influenza A virus infections [16,24,28].

The detection rate of co-infections was similar to previously reported multiplex PCR studies [5,14-17,28,29]. It was observed that higher percentage of children with mixed infections had severe and very severe ALRI as compared to ALRI. Previous studies have shown that co-infection with different respiratory viruses might lead to a more severe disease [31] or multiple viruses have been detected from patients with severe disease [32].

ALRI caused by RSV was more common in younger children as reported previously [28]. RSV and hMPV were associated with bronchiolitis [28,29,33,34]. PIVs and Influenza viruses were associated with pneumonia similar to previous findings [28,29]. However, the number of all the viral detections except RSV was too few to comment on the association of the virus with bronchiolitis or pneumonia.

In the present study, RSV was detected during the fall season similar to previously described studies from our laboratory [35-37]. The rest of the virus identifications were few and their seasonality cannot be commented upon.

## Conclusion

In conclusion, we report a simplified multiplex PCR for the detection of seven respiratory viruses in samples from children with ALRI. This assay was found to be more sensitive, less time consuming and economical than virus isolation. Multiplex PCR format allowed the detection of co-infections which cannot be done using monoplex PCR or culture as shown in the present study.

## Methods

### Patient Specimens

Between April 2005 and March 2007, nasopharyngeal aspirates (NPAs) were collected from children ≤ 5 years of age with ALRI, severe ALRI and very severe ALRI as per WHO criteria [38] and are shown in Table 7.

**Table 7:** Classification of ARLI, severe ALRI and very severe ALRI in children from 2 months to 5 years of age

**Signs**	**Classify as**
Fast breathing as per following criteria according to age	ALRI
-- age less than 2 months: ≥ 60/minute	
-- age 2–11 months: ≥ 50/minute	
-- age 1–5 years: ≥ 40/minute.	
	
Above symptoms with:	Severe ALRI
-- Chest indrawing	
-- Stridor	
-- Nasal flaring	
-- Grunting	
Symptoms of severe pneumonia with:	Very severe ALRI
-- central cyanosis	
-- inability to breastfeed or drink	
-- vomiting everything	
-- convulsions, lethargy or unconsciousness	
-- severe respiratory distress.	

Adapted from POCKET BOOK of Hospital Care for Children Guidelines for the Management of Common Illnesses with limited resources

ISBN 92 4 154670 0 (NLM classification: WS 29)

The children were either seen at the Outpatient Department (OPD) or admitted to the Pediatric Ward of All India Institute of Medical Sciences (AIIMS) Hospital, New Delhi, India. The demographic profile of child, clinical symptoms and risk factors were recorded in a predesigned proforma. NPAs were collected and processed as described earlier [39].

### Standard strains of viruses

Standard strains of 9 viruses namely human respiratory syncytial virus (A2 and 18537), PIV1 (Washington/1964), PIV2 (Greer), PIV3 (D10025), influenza A {H1N1 (A/New Caledonia/20/99) and H3N2 (A/Panama/2007/99)} and B viruses and human metapneumovirus hMPV (Can 97–83) were cultured in Hep-2, MDCK and LLCMK-2 cells as described elsewhere [39-41].

### RNA extraction

RNA was extracted from standard strain of the virus by guanidinium thiocyanate method [42] and 500 μl of NPA using RNeasy kit (Qiagen, GmBH, Germany) described previously [39].

### cDNA synthesis

cDNA synthesis was optimized using 5–20 units of AMV-RT enzyme, 500 ng -1000 ng random hexamer primer (PdN_6_), 0.1–2 mM dNTPs, 4–8 units of RNAsin (all reagents from Promega, Madison, WI, USA) and 5–10 μl of RNA in a 25 μl reaction volume.

### Primer Designing

For RSV, PIVs and hMPV primers were designed from nucleocapsid region and for Influenza (A and B) from the matrix region using sequences available in GenBank, using program OLIGO (Molecular Biology Insights, Cascade, CO, USA, ) and oligonucleotide *T*m calculator (). The sequence of all the seven sets of primers and nested primers for RSV group A and B are shown in Table 8.

**Table 8:** Sequences of oligonucleotides used for detection of viruses in the study

**Target gene**	**Primer**	**Position (nucleotide)**	**Sequence (5' to 3')**	**Amplicon size**
RSV N gene	RSVNF	52–71 bp relative to RSV A (U39961) and RSV B (AF013254)	CTGTCATCCAGCAAATACAC	683 bp
	RSVNR	711–734 bp relative to RSV A (U39961) and RSV B (AF013254)	ACCATAGGCATTCATAAACAATC	
PIV1 N gene	PIV1NF	64–89 bp primer location was relative to NC003461, Washington 1964 strain (Osiowy C 1998)	TCTGGCGGAGGAGCAATTATACCTGG	84 bp
	PIV1NR	122–147 bp primer location was relative to NC003461, Washington 1964 strain (Osiowy C 1998)	ATCTGCATCATCTGTCACACTCGGGC	
PIV2 N gene	PIV2NF	221–242 bp primer location was relative to AF533012, Greer strain	GATGACACTCCAGTACCTCTTG	197 bp
	PIV2NR	395–416 bp primer location was relative to AF533012, Greer strain	GATTACTCATAGCTGCAGAAGG	
PIV3 N gene	PIV3NF	439–465 bp primer location was relative to D10025 strain	GATCCACTGTGTCACCGCTCAATACC	266 bp
	PIV3NR	680–705 bp primer location was relative to D10025 strain	CTGAGTGGATATTTGGAAGTGACCTGG	
hMPV N gene	hMPVNF	79–104 bp primer location was relative to hMPV 00–1 (AF371337) strain (Banerjee *et al*., 2007)	AAGCATGCTATATTAAAAGAGTCTCA	440 bp
	hMPVNR	496–518 bp primer location was relative to hMPV 00–1 (AF371337) strain (Banerjee *et al*., 2007)	ATTATGGGTGTGTCTGGTGCTGA	
RSV N gene (nested primers)	RSVAF	156–180 bp primer location was relative to RSV A (U39961) strains	AAGCAAATGGAGTGGATGTAACAAC	260 bp
	RSVAR	532–554 bp primer location was relative to RSV A (U39961) strains	CTCCTAATCACAGCTGTAAGACCCA	
	RSVBF	135–160 bp primer location was relative to RSV B (AF013254) strain	CAAACTATGTGGTATGCTATTAATCA	328 bp
	RSVBR	463–486 bp primer location was relative to RSV B (AF013254) strain	ACACAGTATTATCATCCCACAGTC	
Influenza A matrix gene	Inf AF	119–140 bp primer location was relative to NC003150 (A/New Caledonia/20/99) and NC032261 (A/Panama/2007/99)	AGGYWCTYATGGARTGGCTAAAG	105 bp
	Inf AR	204–223 bp primer location was relative to NC003150 (A/New Caledonia/20/99) and NC032261 (A/Panama/2007/99)	GCAGTCCYCGCTCASTGGGC	
Influenza B matrix gene	Inf BF	54–76 bp primer location was relative to CY018638 strain	GGAGAAGGCAAAGCAGAACTAGC	503 bp
	Inf BR	531–554 bp primer location was relative to CY018638 strain	CCATTCCATTCATTGTTTTTGCTG	
GAPDH primers	GAPDH1	Gueudin *et al*., 2003	TCA TCC ATG ACAACT TTG GTA TCG TG	564 bp
	GAPDH1	Gueudin *et al*., 2003	CTC TTC CTC TTG TGCTCT TG	

Y, W, R, S are wobbles for C/T, A/T, A/G and G/C

### PCR standardization

cDNA was synthesized from pooled RNA of different viruses to generate template for multiplex PCR. Parameters that were optimized included different concentrations of primers, dNTPs, magnesium chloride (MgCl_2_), Taq polymerase, adjuvants (DMSO and glycerol) and cycling conditions for a 25 μl reaction. If RSV was detected then nested PCR was done for typing of RSV into group A or B. All the PCR reactions were conventional block PCR assays, carried out in GeneAmp^® ^PCR System 9700 (Applied Biosystems, USA) using plasticware from Axygen Scientific, USA.

An internal control glyceraldehyde-3-phosphate dehydrogenase (GAPDH) was included to check the presence of inhibitors of the RT-PCR assay.

### Sensitivity and specificity of the Multiplex PCR

The sensitivity of the multiplex PCR assay was determined by TCID_50 _using Reed and Muench method [43]. Inter and intra assay specificity of the primers was tested with RNA extracted from RSV A and B, PIV1–3, Influenza A and B viruses, hMPV, enteroviruses, cytomegalovirus, herpes simplex virus 1 & 2.

### Virus isolation by centrifugation enhanced culture

Virus isolations were done using centrifugation enhanced culture (CEC) followed by indirect immunofluorescence (IIF) as described previously [35].

### Costing methods

Costs are reported in this manuscript using United States dollar values, with 2006 taken as the reference year for reporting unit prices.

### Metrological data

The environmental factors namely rainfall (cm), temperature (°C) and humidity (RH in %) were acquired from the India Meteorological Department, Regional Meteorological Centre, New Delhi, India.

### Statistical analysis

Statistical analysis was carried out using STATA 9.0 (College station, Texas, USA). Data were presented as number or median (Range). Validity of multiplex PCR in comparison to CEC-IIF was assessed using sensitivity (95% CI), specificity (95% CI) and likelihood ratio. The association between clinical features at the time of presentation and virus detection was tested using Chi-square/Fisher's exact test as appropriate and OR (95% CI) was also calculated. A p value of < 0.05 was considered statistically significant.

## Competing interests

The authors declare that they have no competing interests.

## Authors' contributions

PB carried out all the molecular and culture based assays and prepared the manuscript. WMS contributed in analysis for the paper and drafting the manuscript. SKK, CJ and VT clinically analyzed the pediatric patients and collected samples from them. HSC, SK, LD helped in analyzing data, drafting and critical reviewing of the manuscript. SB conceived the idea, helped in analysis of the data, participated in its design and coordination and helped to frame the manuscript. All the authors have contributed to, seen and approved the final submitted version of the manuscript.

## Authors' information

Preeti Bharaj is a PhD scholar from Department of Microbiology, All India Institute of Medical Sciences, Ansari Nagar, New Delhi, 110029, India.
